# Circular RNA circ_0000284 plays an oncogenic role in the progression of non-small cell lung cancer through the miR-377-3p-mediated PD-L1 promotion

**DOI:** 10.1186/s12935-020-01310-y

**Published:** 2020-06-16

**Authors:** Li Li, Qiaohua Zhang, Ke Lian

**Affiliations:** Medical Oncology Dept.3, Shanxi Bethune Hospital, Shanxi Academy of Medical Science, No. 99 Longcheng Street, Taiyuan, Shanxi China

**Keywords:** Circ_0000284, Non-small cell lung cancer, miR-377-3p, PD-L1

## Abstract

**Background:**

Circular RNAs (circRNAs), a subgroup of non-coding RNAs, are recognized as pivotal mediators in various types of cancers. CircRNA_0000284 (circ_0000284) was manifested to participate in the development of non-small cell lung cancer (NSCLC). The novel functional mechanism of circ_0000284 in NSCLC was investigated in our current study.

**Methods:**

We exploited quantitative real-time polymerase chain reaction (qRT-PCR) to analyze the relative RNA (circRNA, miRNA and mRNA) expression. The assessment of cell proliferation and colony formation was executed by Cell Counting Kit-8 (CCK-8) and colony formation assay, respectively. Transwell assay was implemented to examine cell migration and invasion. All protein levels were assayed using western blot. The role of circ_0000284 in vivo was evaluated via xenograft model. The target relation was estimated by dual-luciferase reporter and RNA immunoprecipitation (RIP) assays.

**Results:**

As for the biological characterization, circ_0000284 was highly stable and localized in the cytoplasm. Circ_0000284 was up-regulated in NSCLC and could predict poor prognosis of NSCLC patients. Both in vitro and in vivo, down-regulation of circ_0000284 refrained tumorigenesis of NSCLC. Besides, microRNA-377-3p (miR-377-3p) was a miRNA target of circ_0000284, and targeted programmed death-ligand 1 (PD-L1). Circ_0000284 was a cancer-promoting circRNA in NSCLC via regulating the miR-377-3p/PD-L1 axis.

**Conclusion:**

Thus, our results unraveled that circ_0000284 facilitated the progression of NSCLC by up-regulating the PD-L1 expression as a competing endogenous RNA (ceRNA) of miR-377, possibly developing a different perspective in understanding the molecular pathogenesis of NSCLC.

## Highlights

1. Circ_0000284 is more stable than linear HIPK3 mRNA and locates in cytoplasm.
2. The up-regulation of circ_0000284is found in NSCLC and related to the low survival of NSCLC patients.
3. Knockdown of circ_0000284 retards tumorigenesis of NSCLC in vitro and in vivo.
4. Circ_0000284 targets miR-377-3p and miR-377-3p targets PD-L1.
5. Circ_0000284 acts as an oncogene in NSCLC through the miR-377-3p/PD-L1 axis.

## Background

Up to 2018, lung cancer still holds the first position in human cancer associated with death, according to cancer statistics from 185 countries around the world [1]. Non-small cell lung cancer (NSCLC), a subtype of lung cancer, makes up an extremely large proportion in lung cancer-related deaths [2]. With the increasing perception of the molecular basis in NSCLC progression, molecule alterations are considered to be correlated with the treatment effect, and targeted therapy goes ahead to stand in the therapies of NSCLC [3, 4]. Non-coding RNAs (ncRNAs), a class of molecules without protein-coding ability, have attracted considerable attention in NSCLC research due to their therapeutic implications [5, 6].

As regards circular RNAs (circRNAs), a subtype of ncRNAs, their specific covalent closed-loop structures are formed by pre-messenger RNAs (pre-mRNAs) backsplicing. And emerging evidence indicated their prognostic and therapeutic responses in NSCLC [7]. For instance, Wang et al. announced that circPRMT5 expedited tumor growth in NSCLC [8]. Similarly, Tan et al. expounded the promoted effects of circRNA F-circEA-4a on migration and invasion of NSCLC [9]. Circ_0000284 (circHIPK3) is derived from the exon2 of HIPK3 gene with the length of 1099 bp, and acts as sponges of many microRNAs (miRNAs) [10]. Latest reports have confirmed that the upregulation of circHIPK3 in NSCLC and its involvement in cellular behaviors [11]. But the functional mechanism of circ_0000284 still needs to be further explored.

Cumulative research information suggested the involvement of miRNAs, belong to ncRNAs with short nucleotide length, in the development and treatment of NSCLC, such as miR-1296a [12], miR-593-5p [13], miR-300 [14], and so on. Issued studies have proved that miR-377-3p acted as an anti-cancer factor in NSCLC [15] and a target of circ-PRMT5 [8], but the correlation between miR-377-3p and circ_0000284 is still obscure. Moreover, miRNAs work as post-transcriptional regulators in various cancers by mediating gene expression after combining with the 3′-untranslated regions (3′UTRs) of target mRNAs [16]. Programmed death-ligand 1 (PD-L1) is an immune checkpoint protein to inhibit tumor immune response and contribute to cancer progression [17]. PD-L1 was shown to be upregulated and involved in the NSCLC metastasis [18, 19]. Also, the relationship between miR-377-3p and PD-L1 remains undiscovered.

Currently, we laid emphasis on the target correlation between circ_0000284 and miR-377-3p as well as miR-377-3p and PD-L1, with an expect of clarifying the regulatory network among these three molecules in NSCLC evolution.

## Materials and methods

### Clinic samples collection

After the written informed consents was signed by 60 NSCLC patients, a total of 60 pairs of NSCLC tissues and normal para-tumor tissues (neighboring the tumor margin) were collected after their pneumonectomy at Shanxi Bethune Hospital. All tissues were snap-frozen in a − 80 °C ultra-low temperature refrigerator for conserving. Besides, these samples contained I − II (n = 34) and III − IV (n = 26) stages as per the classification of the American Joint Committee on Cancer criteria (AJCC) [20], and there were 24 samples with lymphoid node metastasis and 26 samples with no metastasis. Under the condition of acquiring approval from the Ethics Committee of Shanxi Bethune Hospital, our study was launched.

### Cell culture and actinomycin D treatment

In this research, human normal lung fibroblast cell line (MRC-5) and NSCLC cell lines (A549 and H82) were purchased from American Type Culture Collection (ATCC, Manassas, VA, USA) and maintained in specific basic medium complemented with 10% fetal bovine serum (FBS) and 1% penicillin–streptomycin (Gibco, Carlsbad, CA, USA) in the humid environment containing 5% CO_2_ at 37 °C. Dulbecco’s modified eagle medium (DMEM), F-12K and Roswell Park Memorial Institute-1640 (RPMI-1640) from Gibco were used as the basic medium for MRC-5, A549 and H82 cells, respectively. For the inhibition of transcription, 2 μg/mL actinomycin D (Millipore, Billerica, MA, USA) was added to the F-12 K medium to culture A549 cells for 0 h, 6 h, 12 h, 18 h and 24 h.

### Quantitative real-time polymerase chain reaction (qRT-PCR)

In the beginning, total RNA from NSCLC tissues and cells needed to be isolated through Trizol (Beyotime, Shanghai, China). And the nuclear and cytoplasmic RNAs were extracted from cells via a PARIS™ Kit (Invitrogen, Carlsbad, CA, USA). For Ribonuclease R (RNase R) treatment, 2 μg total RNA was incubated with 3 U/μg of RNase R (Epicentre Technologies, Madison, WI, USA) at 37 °C for 60 min. Then RNA was used for synthesizing the complementary DNA (cDNA) using the BeyoRT™ II cDNA synthesis kit (Beyotime). Subsequently, qRT-PCR was carried out to amplify the RNA fragments in terms of the explanatory memorandum of BeyoFast™ SYBR Green qPCR Mix (Beyotime). The used primers contained circ_0000284: Forward (F), 5′-TATGTTGGTGGATCCTGTTCGGCA-3′ and Reverse (R), 5′-TGGTGGGTAGACCAAGACTTGTGA-3′; HIPK3 mRNA: F, 5′-ACATTGGAAGAGCATGAGGCAGAGA-3′ and R, 5′-CTGCTGAAAAGCATCACCACAACCA-3′; miR-377-3p: F, 5′-GGGAGGCAGTGTATTGTTA-3′ and R, 5′-GTCGTATCCAGTGCAGGGTCCGAGGT-3′; glyceraldehyde-3-phosphate dehydrogenase (GAPDH): F, 5′-CTCCTCCACATTTGACGCTG-3′ and R, 5′-TCCTCTTGTGCTCTTGCTGG-3′; U6: F, 5′-GTAGATACTGCAGTACG-3′ and R, 5′-ATCGCATGACGTACCTGAGC-3′.GAPDH served as the internal reference of circ_0000284 and HIPK3 mRNA, while U6 was applied to standardize miR-377-3p expression. The data analysis was implemented via the comparative cycle threshold (2^−∆∆Ct^) method.

### Cell transfection

According to the user’s manual, the transfection of different groups in A549 and H82 cells was administrated using Lipofectamine 3000 (Invitrogen, Carlsbad, CA, USA). Short hairpin RNA (shRNA) against circ_0000284 (sh-circ_0000284) was transfected via lentiviral to establish the stably expressed cell lines, with sh-NC as the negative control. MiR-377-3p mimic (miR-377-3p)/miRNA mimic (miR-NC), miR-377-3p inhibitor (anti-miR-377-3p)/miRNA inhibitor (anti-NC) and small interfering RNA (siRNA) targeting PD-L1 (si-PD-L1)/siRNA negative control (si-NC) were directly transfected into cells. All these oligonucleotides were bought from GenePharma (Shanghai, China). In addition, the sequence of circ_0000284 was cloned into the pcDNA3.1 (+) CircRNA Mini vector (YuBo Biotech, Shanghai, China) for overexpressing circ_0000284. The recombinant vector was named as circ_0000284 and the negative control was displayed as vector.

### Cell Counting Kit-8 (CCK-8) assay

100 μL cell suspensions (2 × 10^3^ cells) were plated into each well of 96-well plates for 16 h. At 72 h post-transfection, cells were mixed with CCK-8 reagent (Beyotime, 10 μL/well) to incubate for 2 h. The absorbance of each well was assayed at the wavelength of 450 nm by a microplate reader (Thermo Fisher Scientific, Waltham, MA, USA).

### Colony formation assay

Cells were seeded at a density of 500 cells per well in a six-well plate. About 2 weeks later, macroscopic colonies were emerged, and then phosphate buffered saline (PBS; Sigma, St. Louis, MO, USA) was used for washing cells twice. After the fixation of methanol (Sigma) and the dyeing of crystal violet (Sigma), the colony cells were counted via a microscope (Thermo Fisher Scientific).

### Transwell assay

A transwell 24-well chamber (Corning Inc., Corning, NY, USA) was exploited for the detection of migration and invasion. Briefly, cell suspension (2 × 10^4^ cells) in the serum-free medium was inoculated into the upper chamber, along with the addition of medium containing 10% FBS in the lower chamber. After incubation for 24 h, cells could pass through the filter due to the attraction of the medium in the lower chamber. The passed cells were fixed and stained by methanol and crystal violet, and then the number was counted under an inverted microscope (Olympus, Tokyo, Japan). Different from the examination of migration, the upper transwell chamber must be coated with matrigel (Corning Inc.) before the inoculation of cells in invasion detection.

### Western blot

After the protein extraction and quantification using the radio immunoprecipitation assay (RIPA) buffer and BCA protein determination kit (Sigma), 40 µg proteins were separated by using sodium dodecyl sulfate–polyacrylamide gel electrophoresis (SDS-PAGE). Subsequently, we transferred the proteins onto the polyvinylidene fluoride membranes (Millipore) and Western Blocker™ solution (Sigma) was employed for preventing the non-specific combination, followed by the incubation of primary antibodies (overnight at 4 °C) and secondary antibody (at the room temperature for 1 h). Ultimately, the immune binding was discerned using the ECL Western HRP Substrate (Millipore), and analyzed with ImageLab software version 4.1 (Bio-Rad, Hercules, CA, USA). The antibodies were from Abcam (Cambridge, UK): anti-ki67 (ab16667, 1:1000), anti-E-cadherin (ab40772, 1:1000), anti-Fibronectin (ab32419, 1:1000), anti-matrix metalloproteinase 9 (anti-MMP9; ab219372, 1:1000), anti-PD-L1 (ab228415, 1:1000), internal control anti-GAPDH (ab128915, 1:3000) and anti-rabbit IgG/HRP-linked secondary antibody (ab205718, 1:5000).

### Xenograft tumor assay

BALB/c female nude mice (6 weeks old, n = 10) were purchased from Shanghai Animal Experimental Center (Shanghai, China) and divided into two groups (sh-circ_0000284 and sh-NC) at random. The xenograft model was established by the administration of subcutaneous injection into mice with A549 cells stably transfected with sh-circ_0000284 or sh-NC. Weekly, the tumor size was measured and tumor volume (length × width^2^/2) was recorded. 35 d later, mice were euthanized according to institutional ethics/animal care guidelines/protocols, then tumors were immediately taken out and weighed. And circ_0000284 expression was analyzed by qRT-PCR after RNA extraction from tumors. Our animal experiment was endorsed by the Animal Ethics Committee of Shanxi Bethune Hospital.

### Dual-luciferase reporter assay

Vector construction was carried out after the amplification of circ_0000284 and 3′UTR of PD-L1, including the fragments of wild-type (WT) containing the target region for miRNA, and mutant-type (MUT) indicating that the target bites in WT were mutated into the same nucleobase with miRNA. When their expression was detected in the pGL3 vector (Promega, Madison, WI, USA), suggesting that the recombinant reporters WT-circ_0000284/MUT-circ_0000284 and WT-PD-L1 3′UTR/MUT-PD-L1 3′UTR were obtained. In addition, WT-HIPK3 and MUT-HIPK3 plasmids were constructed as the above description. WT and MUT vectors were transfected into A549 and H82 cells with miR-377-3p or miR-NC, respectively. Eventually, the luciferase activity in cell lysate was analyzed through the dual-luciferase assay system (Promega) in the light of the supplied instruction book. The firefly luciferase activity was normalized by renilla activity.

### RNA immunoprecipitation (RIP) assay

Based on the operating procedures of the Magna RIP™ kit (Millipore), RIP assay was discreetly performed. In brief, cells were harvested in RIP lysis buffer, and incubated with antibodies-covered magnetic beads. The anti-immunoglobulin G (Anti-IgG) was used as the negative control for anti-against argonaute2 (Anti-Ago2). Following the protein removal using proteinase K (Sigma) from magnetic beads and RNA isolation, qRT-PCR was exploited to examine the levels of circ_0000284 and miR-377-3p.

### Statistical analysis

All experiments were independently performed three times with three biological replications each time. Data were shown as the mean ± standard deviation (SD). SPSS 22.0 and GraphPad Prism 7 were employed for statistical processing. The survival curve was formed by the Kaplan–Meier plot, and analyzed by the log-rank test. Difference analysis was executed via the Student’s *t* test and one-way analysis of variance (ANOVA) followed by Tukey’s test. *P *< 0.05 was defined as a significant difference.

## Results

### Biological characterization of circ_0000284 in NSCLC cells

**Fig. 1:**
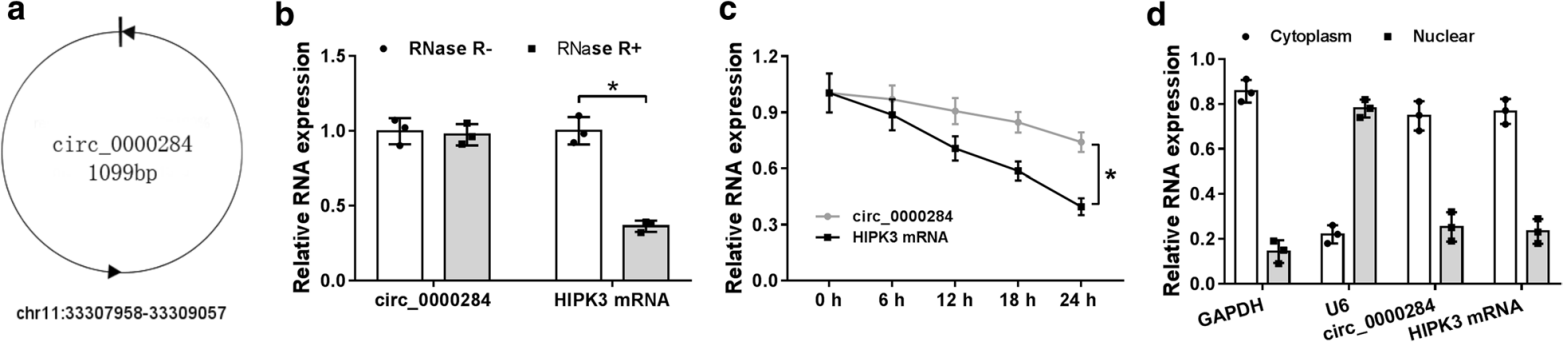
Biological characterization of circ_0000284 in NSCLC cells. **a** The basic information of circ_0000284 was exhibited according to circBank. **b**, **c** The expression levels of circ_0000284 and HIPK3 mRNA were determined by qRT-PCR after RNase R (B) or actinomycin D (**c**) treatment. **d** The location analysis of circ_0000284 in A549 cells was conducted using qRT-PCR by comparison with HIPK3, GAPDH and U6. All experiments were independently performed three times with N value = 3. *P < 0.05

Certainly, the properties of circ_0000284 should be analyzed. After the searching in circBank, we cognized that circ_0000284 was originated from HIPK3 gene with the position of chr11: 33,307,958-33,309,057 and formed by backsplicing with the length of 1099 bp (Fig. 1a). Whereafter, the characterization of circ_0000284 was determined in A549 cells. For the stability of circ_0000284, the expression of circ_0000284 was slightly decreased, while HIPK3 mRNA level was reduced by 60% after treatment of RNase R for 1 h (Fig. 1b) and actinomycin D for 24 h (Fig. 1c), indicating that circ_0000284 was much more stable than linear HIPK3. For the localization of circ_0000284, we observed the circ_0000284 level was abundantly enriched in cytoplasm as well as HIPK3 gene, with GAPDH and U6 as respective controls for cytoplasm and nucleus (Fig. 1d), which demonstrated that circ_0000284 was mainly localized in the cytoplasm.

### Circ_0000284 expression was aberrantly increased in NSCLC and this up-regulation was associated with the low survival in NSCLC patients

**Fig. 2:**
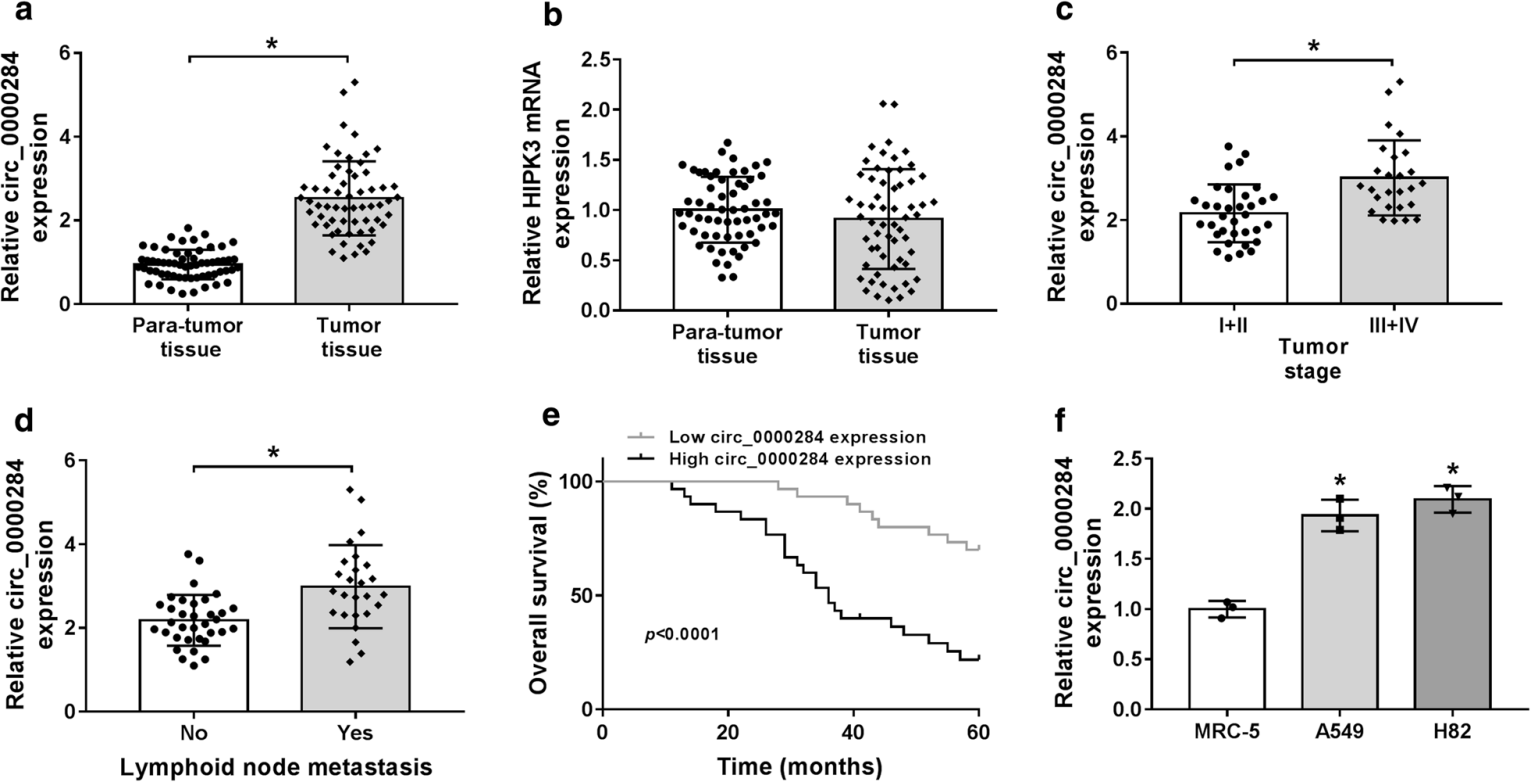
Circ_0000284 expression was aberrantly increased in NSCLC and this up-regulation was associated with the low survival in NSCLC patients. **a**, **b** Circ_0000284 (**a**) and HIPK3 mRNA (**b**) levels were detected via qRT-PCR in NSCLC and normal tissues. **c**, **d** The qRT-PCR was conducted for circ_0000284 expression analysis in NSCLC tissues of I + II and III + IV stages (**c**) and NSCLC tissues with or without lymphoid node metastasis (**d**). **e** Survival curve of patients was generated through a Kaplan–Meier plot and analyzed by the log-rank test. **f** The relative level of circ_0000284 in MRC-5, A549 and H82 cells was assayed through qRT-PCR. All experiments were independently performed three times with N value = 3. *P < 0.05

Then, the relative expression of circ_0000284 in NSCLC was examined by qRT-PCR. As Fig. 2a illustrated, the expression level of circ_0000284 in the collected NSCLC tissues was higher than that in the corresponding para-tumor tissues. But the mRNA expression of its host gene HIPK3 wasn’t differentially expressed in NSCLC and normal tissues (Fig. 2b). After tumor classification, circ_0000284 was also found to be up-regulated by approximate 50% in III + IV stages relative to I + II stages among 60 NSCLC tissues (Fig. 2c). Meanwhile, compared with no-metastasis tissues, circ_0000284 was increased by 50% in metastatic NSCLC tissues, which demonstrated that up-regulated circ_0000284 might be related to lymphoid node metastasis (Fig. 2d). It is interesting that the overall 5-year survival was signally decreased in NSCLC patients over-expressed circ_0000284 (n = 30, containing a missing patient) compared to those patients with low circ_0000284 expression (n = 30) (Fig. 2e), implicating the correlation between the up-regulated circ_0000284 and low survival. Also, the elevation of circ_0000284 was verified in NSCLC cells (A549 and H82) using normal MRC-5 cells as the reference (Fig. 2f). These results hinted the high expression of circ_0000284 was relevant to the NSCLC progression.

### Circ_0000284 was specifically knocked down by shRNA in NSCLC cells

**Fig. 3:**
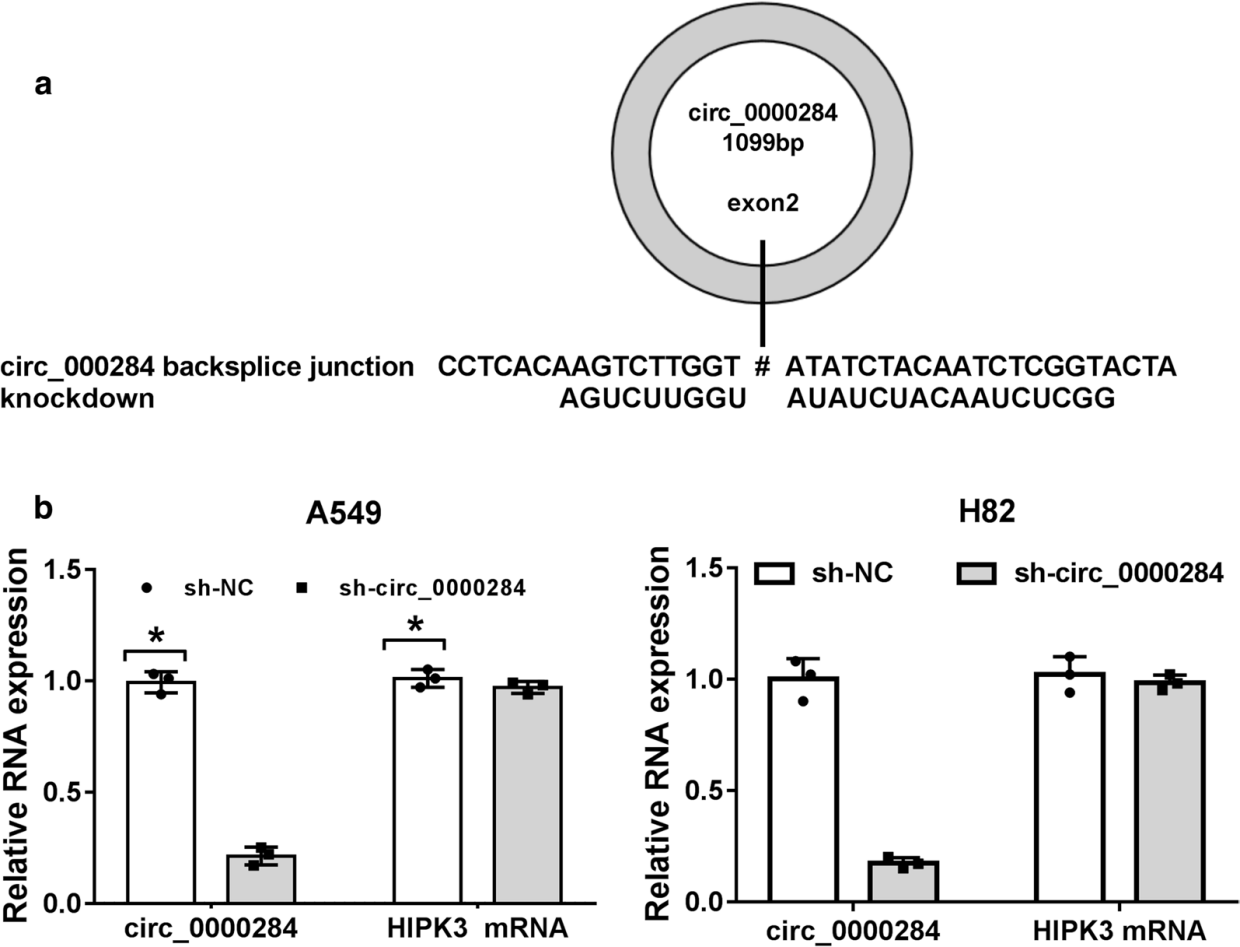
Circ_0000284 was specifically knocked down by shRNA in NSCLC cells. **a** Circ_0000284 knockdown sites in the backsplice junction. **b** The qRT-PCR was exploited to evaluate the expression levels of circ_0000284 and HIPK3 mRNA. *P < 0.05

To analyze the biological role of circ_0000284 in NSCLC, shRNA targeting circ_0000284 was used for its expression knockdown. The specific shRNA was designed to target the backsplice junction in the exon 2 of the HIPK3 gene (Fig. 3a). As shown in Fig. 3b, the shRNA-mediated circ_0000284 knockdown was quite evident in both A549 and H82 cells but its linear transcript HIPK3 expression was not obviously affected by sh-circ_0000284. Hence, the shRNA-mediated knockdown of circ_0000284 was highly specific.

### Knockdown of circ_0000284 impeded tumorigenesis of NSCLC in vitro and in vivo

**Fig. 4:**
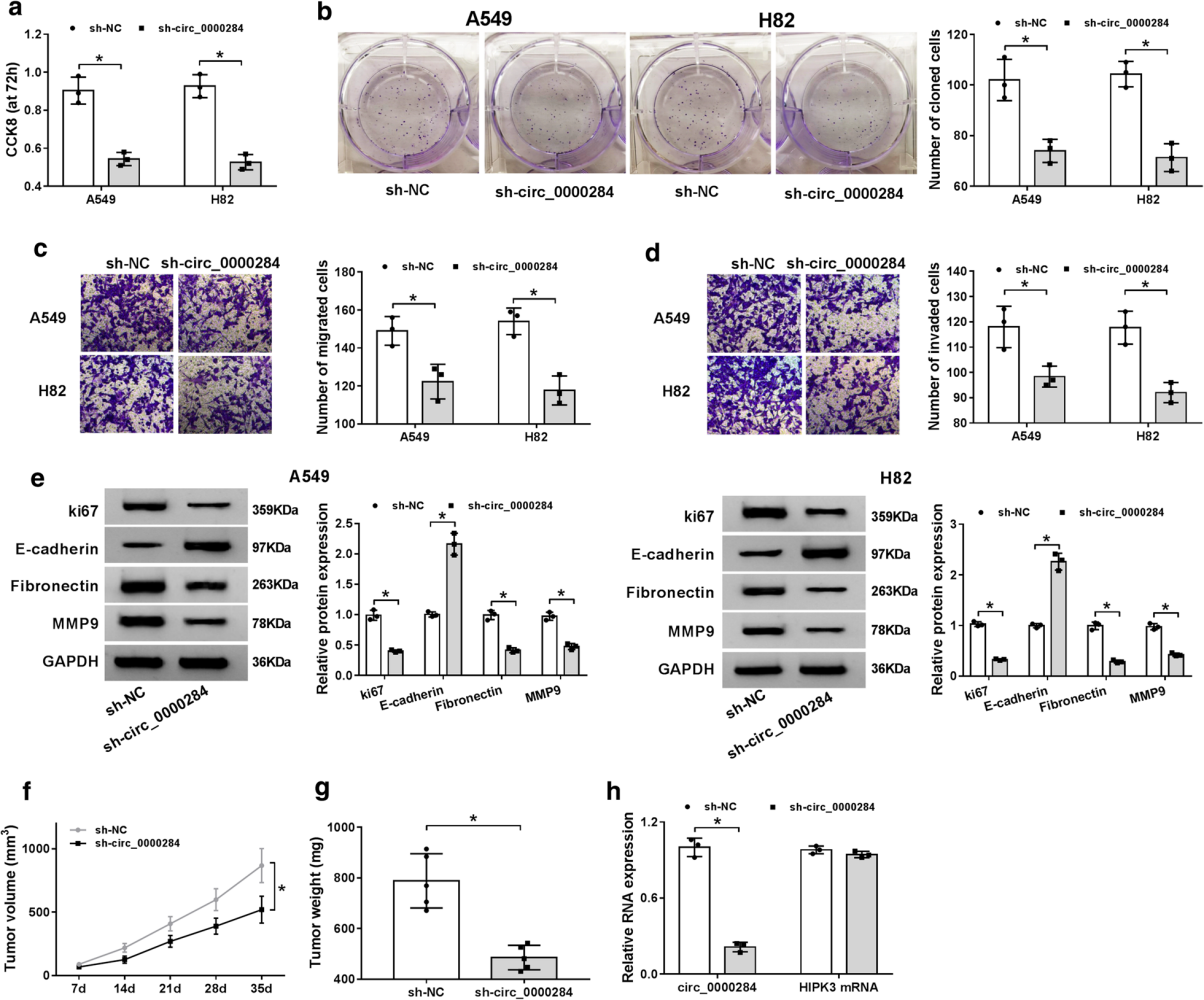
Knockdown of circ_0000284 impeded tumorigenesis of NSCLC in vitro and in vivo. A549 and H82 cells were severally transfected with sh-NC or sh-circ_0000284. **a**–**d** The measurement of cell proliferation (**a**), colony formation (**b**) and migration/invasion (**c**, **d**) was implemented with respective CCK-8, colony formation assay and transwell assay. **e** The proliferation and metastasis-related proteins were determined using western blot. **f**, **g** The volume (**f**) and weight (**g**) of tumors in sh-NC and sh-circ_0000284 groups were measured. **h** Circ_0000284 and HIPK3 mRNA expression levels in tumors of mice were analyzed by qRT-PCR. All experiments were independently performed three times with N value = 3. *P < 0.05

After transfection of sh-circ_0000284 for 72 h, cell proliferation of A549 and H82 cells was obviously suppressed by 50% in contrast to sh-NC group (Fig. 4a). The same inhibition was observed in the detection of colony formation after circ_0000284 knockdown (Fig. 4b). In transwell assay, the migrated (Fig. 4c) and invaded (Fig. 4d) cells in sh-circ_0000284 transfection group were fewer than these in sh-NC group. Afterwards, we examined the protein levels of some important markers in these cellular processes, including ki67 (a well-known proliferation marker) [21] and metastasis-related indictors (anti-metastatic E-cadherin, pro-metastatic Fibronectin and MMP9) [22, 23]. As the depiction of Fig. 4e, the repression of circ_0000284 expression resulted in the conspicuous lessening of ki67, Fibronectin and MMP9, and promotion of E-cadherin, insinuating that sh-circ_0000284 inhibited the proliferation and metastasis of NSCLC cells. Moreover, the experiment in vivo manifested that the introduction of sh-circ_0000284 distinctly reduced tumor volume (Fig. 4f) and weight (Fig. 4g) in mice compared to sh-NC. And the expression of circ_0000284 was indeed inhibited by 80% in sh-circ_0000284 group making a contrast with sh-NC group, while this inhibition was almost not observed on HIPK3 mRNA level (Fig. 4h). All in all, knockdown of circ_0000284 (not HIPK3) impeded the NSCLC tumorigenesis in vitro and in vivo.

### Circ_0000284 negatively regulated the expression of miR-377-3p

**Fig. 5:**
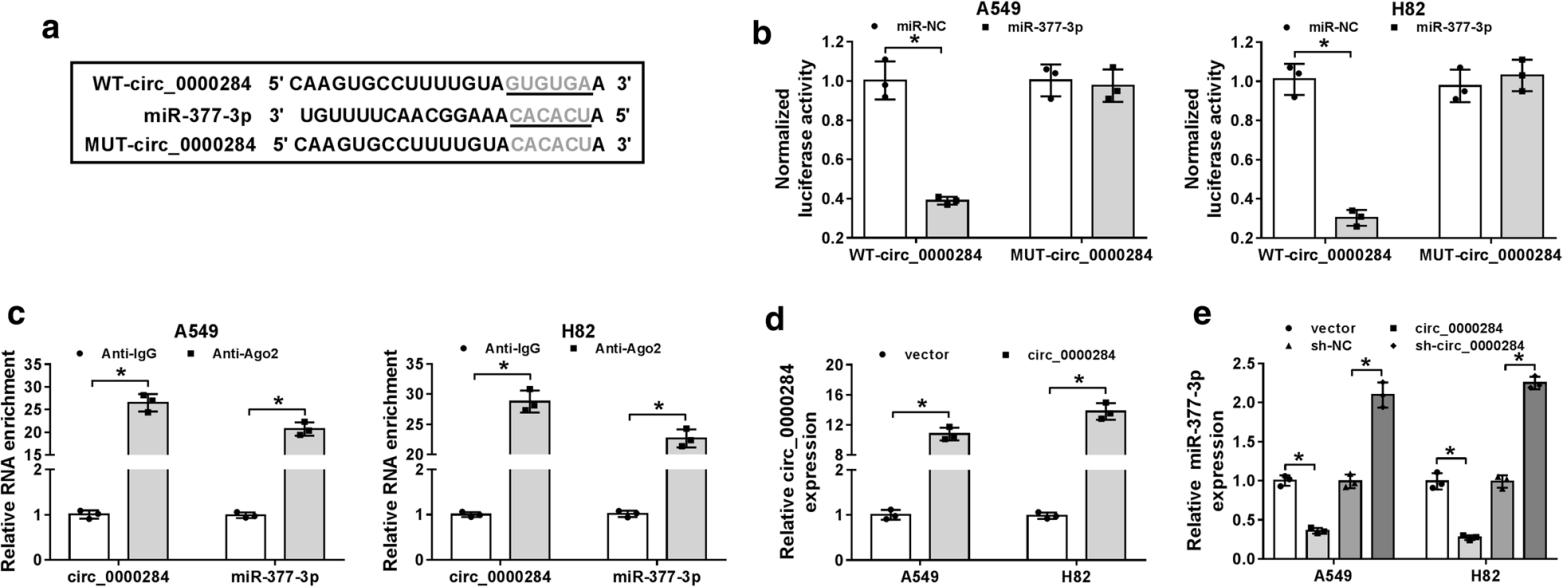
Circ_0000284 negatively regulated the expression of miR-377-3p. **a** Circular RNA Interactome was applied for the bioinformatics analysis of circ_0000284 and miR-377-3p. **b**, **c** The binding between circ_0000284 and miR-377-3p was testified by dual-luciferase reporter assay (**b**) and RIP assay (**c**). **d** The overexpression efficiency of circ_0000284 in A549 and H82 cells was assessed via qRT-PCR. **e** The qRT-PCR was administrated to estimate the effects of circ_0000284 up-regulation or down-regulation on the expression of miR-377-3p. All experiments were independently performed three times with N value = 3. *P < 0.05

Through the prediction of Circular RNA Interactome, the nucleotide-binding sites of miR-377-3p were noticed in the sequence of circ_0000284 (Fig. 5a). For the sake of confirming the actual binding between circ_0000284 and miR-377-3p, the dual-luciferase reporter assay was carried out firstly. In four groups, only WT-circ_0000284 and miR-377-3p co-transfection exerted the marked decline of normalized luciferase activity in A549 and H82 cells, which implied the combination of circ_0000284 and miR-377-3p (Fig. 5b). However, the luciferase signal of WT-HIPK3 plasmid was not decreased as MUT-HIPK3 plasmid after the overexpression of miR-377, suggesting that HIPK3 couldn’t bind to miR-377 (Additional file 1: Fig. S1A). Simultaneously, RIP assay was also used for proving the attraction of circRNA by miRNA in RNA-induced silencing complex (RISC) using the antibody against Ago2 protein, an indispensable component in RISC [24]. As displayed in Fig. 5c, both circ_0000284 and miR-377-3p were largely enriched by the Anti-Ago2 in comparison to Anti-IgG. And transfection of circ_0000284 successfully heightened the expression of circ_0000284 compared with its control vector (Fig. 5d), but not HIPK3 mRNA expression (Additional file 1: Fig. S1B). Then qRT-PCR results demonstrated that miR-377-3p level was remarkably restrained by the overexpression of circ_0000284, but enhanced by twofold changes after the down-regulation of circ_0000284 (Fig. 5E). Hence, circ_0000284 negatively regulated its target miR-377-3p.

### PD-L1 acted as a molecular target of miR-377-3p

**Fig. 6:**
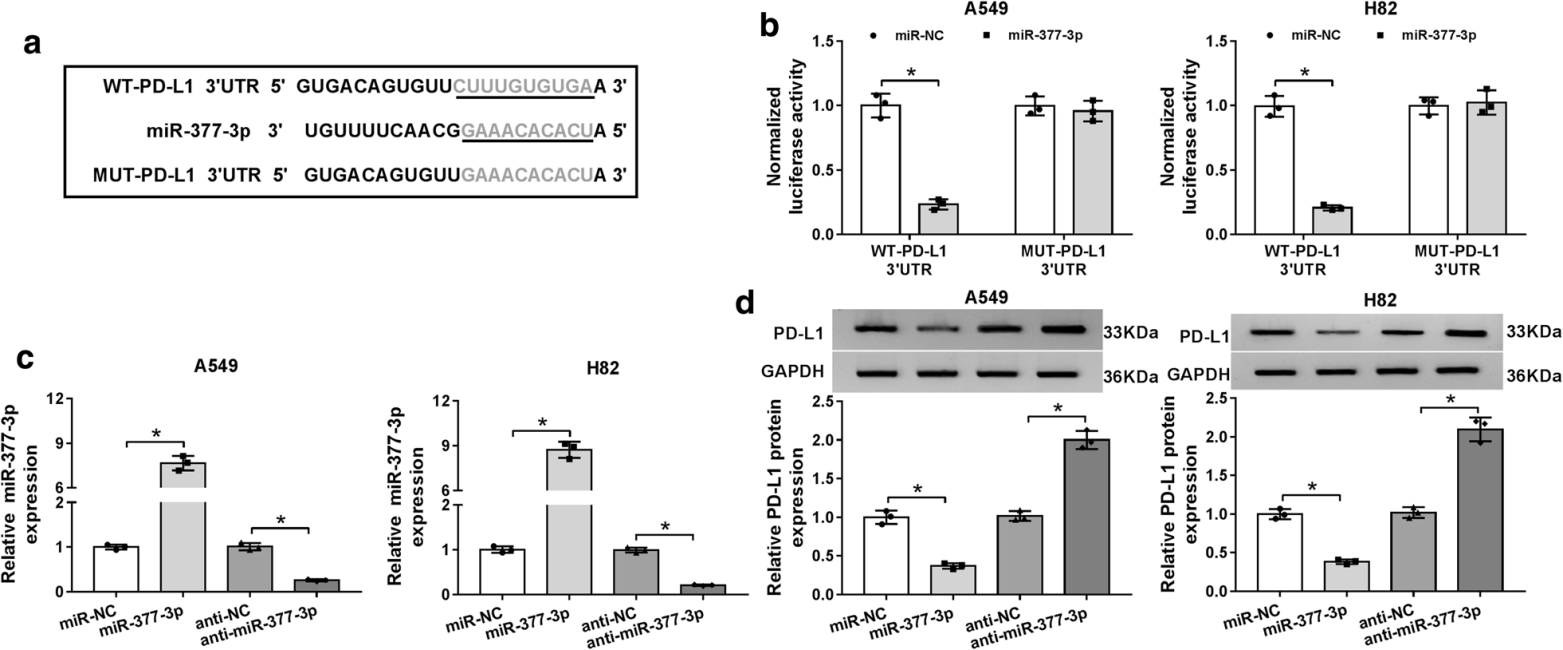
PD-L1 acted as a molecular target of miR-377-3p. **a** The combinative sites between miR-377-3p and 3′UTR of PD-L1 were exhibited after the analysis of Starbase 3.0. **b** Dual-luciferase reporter assay was employed for validating whether miR-377-3p could combine with PD-L1. **c** The efficiencies of miR-377-3p mimic or inhibitor were evaluated using qRT-PCR. **d** PD-L1 protein level was analyzed by western blot in A549 and H82 cells transfected with miR-NC, miR-377-3p, anti-NC or anti-miR-377-3p. All experiments were independently performed three times with N value = 3. *P < 0.05

On behalf of seeking the downstream target of miR-377-3p, Starbase 3.0 online prediction was performed. The results suggested that miR-377-3p could bind to the 3′UTR of PD-L1 presumptively (Fig. 6a). After the implement of dual-luciferase reporter assay, miR-377-3p mimic led to an overt reduction by 75% in luciferase intensity of WT-PD-L1 3′UTR vector, but had no evident suppressive action on MUT-PD-L1 3′UTR vector (Fig. 6b). Whereafter, the effect of miR-377-3p on PD-L1 was explored. After the detection of miR-377-3p expression by qRT-PCR, we found that the overexpression of miR-377-3p and the inhibition of anti-miR-377-3p on miR-377-3p level were successful in A549 and H82 cells (Fig. 6c). Then western blot analysis revealed that PD-L1 protein level of miR-377-3p transfection group was much lower than that of miR-NC group, but it is contrary that miR-377-3p inhibitor caused a simulative effect on PD-L1 protein expression (Fig. 6d). Clearly, PD-L1 was a downstream target of miR-377-3p.

### Circ_0000284 generated the oncogenic function in NSCLC cells via the regulation of miR-377-3p/PD-L1 axis

**Fig. 7:**
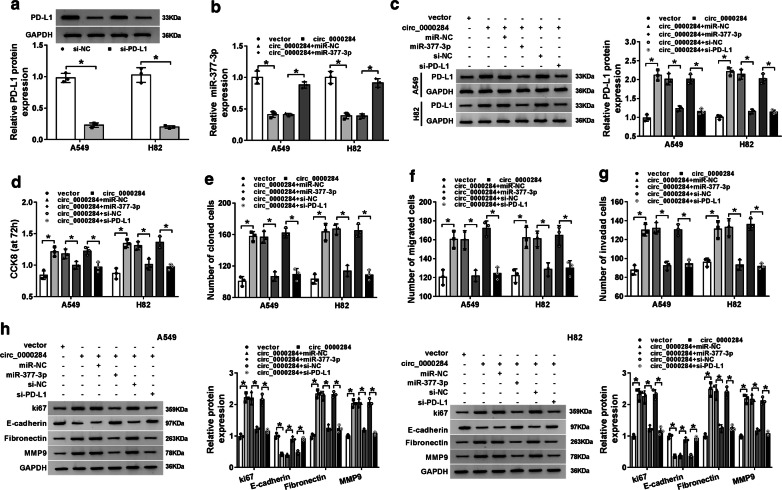
Circ_0000284 generated the oncogenic function in NSCLC cells via the regulation of miR-377-3p/PD-L1 axis. **a** PD-L1 expression was assayed through western blot in A549 and H82 cells transfected with si-NC or si-PD-L1. **b** The examination of miR-377-3p was conducted using qRT-PCR after transfection of circ_0000284, circ_0000284 + miR-377-3p or relative controls. **b** Western blot was applied for measuring the protein level of PD-L1 following the transfection of circ_0000284, circ_0000284 + miR-377-3p, circ_0000284 + si-PD-L1 as well as their controls. **d**, **e** CCK-8 and colony formation assays were used for the respective detection of cell proliferation (**d**) and colony formation ability (**e**). **f**, **g** Cell migration (**f**) and invasion (**g**) were evaluated using transwell assay. **h** The determination of proliferation and metastasis-associated protein levels was performed via western blot. All experiments were independently performed three times with N value = 3. *P < 0.05

We applied siRNA transfection to block the expression of PD-L1, and western blot indicated the interference of si-PD-L1 was notable, in comparison with the si-NC transfection (Fig. 7a). And miR-377-3p mimic could restore the inhibition of circ_0000284 in miR-377-3p expression (Fig. 7b). Furthermore, the overexpression of circ_0000284 led to the corresponding increase of PD-L1 protein level, while this influence was almost abrogated after miR-377-3p up-regulation or PD-L1 inhibition (Fig. 7c). And apparently, the circ_0000284-induced refrained effects on cell proliferation (Fig. 7d), colony formation (Fig. 7e), migration and invasion (Fig. 7f–g) were all relieved after the transfection of miR-377-3p or si-PD-L1. Similarly, the promotion of ki67, Fibronectin, MMP9, and the reduction of E-cadherin triggered by circ_0000284 were also alleviated when miR-377-3p expression was boosted or PD-L1 was suppressed (Fig. 7h). Collectively, the oncogenic function of circ_0000284 in NSCLC cells was achieved by regulating the miR-377-3p/PD-L1 axis (the inhibition of miR-377-3p and promotion of PD-L1).

## Discussion

CircRNAs have been reported to possess the potential for diagnosing and treating NSCLC [6]. Understanding the pathogenesis of NSCLC in the field of circRNAs is forceful for supporting circRNAs as molecular targets for NSCLC treatment. Through a series of experiments, we illuminated that circ_0000284 contributed to the tumorigenesis of NSCLC by promoting PD-L1 expression via sequestering miR-377-3p, developing a novel insight into the pathological mechanism of NSCLC.

HIPK3 (a paralogous kinase) was reported to produce abundant back-spliced products, which were deemed as circRNAs [25]. And circRNAs are not degraded as efficiently as linear mRNAs due to the absence of free 5′ and 3′ ends, and predominantly released to the cytoplasmic environment [25]. Verifiably, circ_0000284 was resistant to RNase R digestion and actinomycin D treatment, which proved its high stability. The cytoplasm localization of circ_0000284 was also identified in our study.

Here, the overexpression of circ_0000284 was considered to be an indicator of NSCLC occurrence and low survival in NSCLC patients. Previously, circ_0000284 (circHIPK3) was found to be up-regulated, and could enhance the tumor development in many other human cancers, such as prostate cancer [26], epithelial ovarian cancer [27], colorectal cancer [28], gastric cancer [29] and glioma [30]. Wang et al. also proclaimed that upregulation of circ_0000284 promoted cell proliferation, migration and invasion abilities in cholangiocarcinoma [31]. In accordance with these reports, knockdown of circ_0000284 brought about the inhibition of NSCLC cellular behaviors (cell proliferation, colony formation, migration and invasion) in this research. In vivo, the low expression of circ_0000284 also impeded tumor growth of NSCLC. Altogether, these data exhibited that the suppression of circ_0000284 might be used as an instrumental therapeutic strategy to retard the progression of NSCLC.

Afterwards, we found that miR-377-3p could be sponged by circ_0000284, and PD-L1 expression was directly inhibited by miR-377-3p. The target relationship between miR-377-3p and circ_0000284 or PD-L1, was firstly be verified. The hypothesis that circRNAs function as competing endogenous RNAs (ceRNAs) of miRNAs to abolish the inhibition of miRNAs on target genes and elevate the gene expression has been widely recognized in various cancers [32]. For example, Jin et al. showed that circ_0102049 served as a sponge of miR-1304-5p to promote MDM2, thereby accelerating the progression of osteosarcoma [33]. Chen et al. alleged that circ_100290 improved cell growth and glycolysis of oral squamous cell carcinoma by acting as a ceRNA for miR-378a to relieve the inhibition of GLUT1 [34]. And the circ-CMPK1/miR-302e/cyclin D1 axis was disclosed in NSCLC [35]. Herein, circ_0000284 was shown to counteract the miR-377-3p-mediated PD-L1 suppression, consequently increasing the expression of PD-L1, which further caused the progression of NSCLC.

## Conclusion

In summary, our present study elucidated that circ_0000284 served as an oncogene in NSCLC by regulating the miR-377-3p/PD-L1 axis. The circ_0000284/miR-377-3p/PD-L1 regulatory axis was beneficial to strengthen the molecular recognition about the pathology of NSCLC. Circ_0000284 up-regulation can be an effective indicator for NSCLC diagnosis, and circ_0000284 down-regulation may have a significant therapeutic value for NSCLC treatment.

## Supplementary information

**Additional file 1: Fig. S1**. HIPK3 could not bind to miR-377 in NSCLC cells. (A) The luciferase activity was analyzed by dual-luciferase reporter system after the co-transfection of WT-HIPK3 or MUT-HIPK3 and miR-377 or miR-NC. (B) HIPK3 mRNA level in A549 and H82 cells transfected with circ_0000284 or vector was determined using qRT-PCR. All experiments were independently performed three times with N value = 3. *P < 0.05.

## Data Availability

All data generated or analyzed during this study are included in this published article.

## Abbreviations

circRNAs: Circular RNAs
circ_0000284: CircRNA_0000284
NSCLC: Non-small cell lung cancer
qRT-PCR: Quantitative real-time polymerase chain reaction
RIP: RNA immunoprecipitation
miR-377-3p: MicroRNA-377-3p
PD-L1: Programmed death-ligand 1
ceRNA: Competing endogenous RNA
ncRNAs: Non-coding RNAs
pre-mRNAs: Pre-messenger RNAs
