# Supplementary figures and images for: Circular RNA circ_0000284 plays an oncogenic role in the progression of non-small cell lung cancer through the miR-377-3p-mediated PD-L1 promotion
Source: Cancer Cell Int. 2020 Jun 16;20:247. doi: 10.1186/s12935-020-01310-y (PMC7298744; doi:10.1186/s12935-020-01310-y)

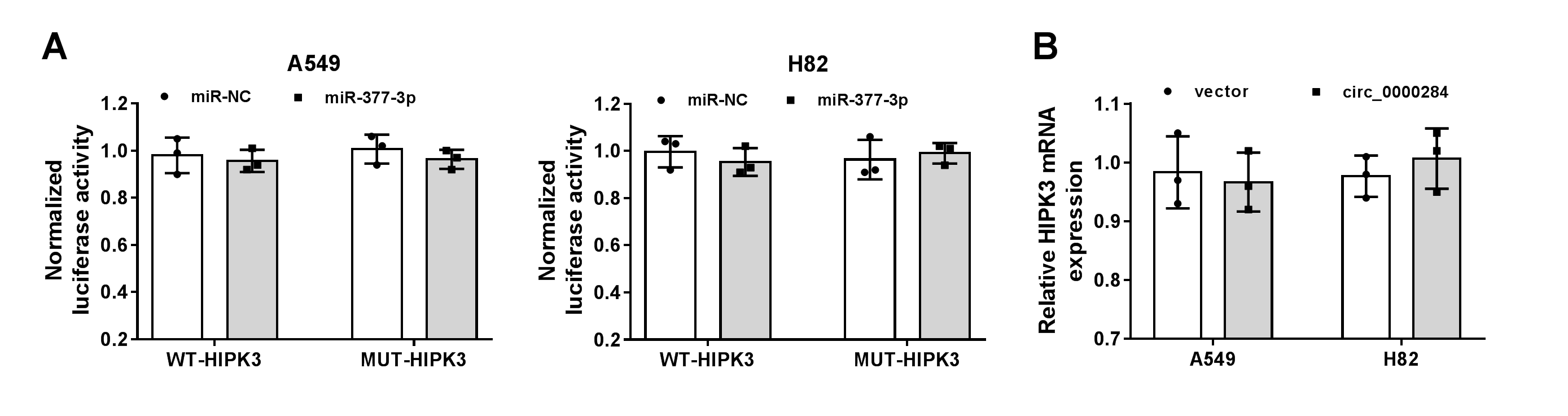

Supplement: Supplementary file 1 — Additional file 1: Fig. S1. HIPK3 could not bind to miR-377 in NSCLC cells. (A) The luciferase activity was analyzed by dual-luciferase reporter system after the co-transfection of WT-HIPK3 or MUT-HIPK3 and miR-377 or miR-NC. (B) HIPK3 mRNA level in A549 and H82 cells transfected with circ_0000284 or vector was determined using qRT-PCR. All experiments were independently performed three times with N value = 3. *P < 0.05. [file 12935_2020_1310_MOESM1_ESM.tif]
